# Acute Effects of Surgical and FFP2 Face Masks on Physiological Responses and Strength Performance in Persons with Sarcopenia

**DOI:** 10.3390/biology10030213

**Published:** 2021-03-11

**Authors:** Domingo Jesús Ramos-Campo, Silvia Pérez-Piñero, Juan Carlos Muñoz-Carrillo, Francisco Javier López-Román, Esther García-Sánchez, Vicente Ávila-Gandía

**Affiliations:** 1 Department of Education, University of Alcalá, 28805 Madrid, Spain; djramos@ucam.edu; 2 Department of Exercise Physiology, Universidad Católica San Antonio de Murcia, 30107 Murcia, Spain; jcmunoz@ucam.edu (J.C.M.-C.); jlroman@ucam.edu (F.J.L.-R.); vavila@ucam.edu (V.Á.-G.); 3 Primary Care Research Group, Biomedical Research Institute of Murcia (IMIB-Arrixaca), 30120 Murcia, Spain; 4 Fundación para la Formación e Invetigación Sanitarias de la Región de Murcia, 30003 Murcia, Spain; garciasanchezesther10@gmail.com

**Keywords:** COVID-19, half-squat, muscle mass, pandemic, resistance training

## Abstract

**Simple Summary:**

Study comparing the use of a surgical mask, FFP2 or none in people with sarcopenia during a resistance training session on strength performance, heart rate, heart rate variability, blood lactate concentration or rating of perceived effort.

**Abstract:**

Due to COVID-19, wearing a face mask to reduce virus transmission is currently mandatory in some countries when participants practice exercise in sports centers. Therefore, the aim of the present study was to analyze the effect of wearing a surgical or FFP2 mask during a resistance training session. Fourteen people with sarcopenia (age: 59.40 ± 5.46 years; weight: 68.78 ± 8.31 kg; height: 163.84 ± 9.08 cm) that participated in the study performed three training sessions in a randomized order: 4 sets of 10 repetitions of a half-squat at 60% of the one-repetition maximum and 90 s of rest between set and were either (a) without a mask (NM), (b) wearing a surgical face mask (SM), and (c) wearing a FFP2 face mask (FFP2). We found that wearing face masks had no effect on strength performance (session mean propulsive velocity (m/s): WM: 0.396 ± 0.042; SM: 0.387 ± 0.037; and FFP2: 0.391 ± 0.042 (*p* = 0.918)). Additionally, no impact of wearing a mask was found on heart rate, heart rate variability, blood lactate concentration (WM: 4.17 ± 1.89; SM: 4.49 ± 2.07; and FFP2: 5.28 ± 2.45 mmol/L (*p* = 0.447)), or rating of perceived exertion. Wearing a surgical or FFP2 face mask during a resistance training session resulted in similar strength performance and physiological responses than the same exercise without a mask in persons with sarcopenia.

## 1. Introduction

Sarcopenia is a major clinical and public health problem in older people, with an overall worldwide prevalence of 10% that is rising [1]. Sarcopenia is a component of fragility syndrome and indicates a significant health issue related to a progressive decline in muscle tissue quality and strength [2]. This progressive and generalized skeletal muscle disorder is associated with increased likelihood of adverse outcomes including falls, fractures, physical disability, and mortality [3]. To date, exercise should be considered a fundamental aspect of the treatment of pathological skeletal muscle mass reduction [2]; it is well-accepted that physical exercise is one of the cornerstones for the prevention and treatment of sarcopenia [4], with resistance training being the most common treatment due to compelling evidence showing that it improves muscle mass, muscle strength, and physical performance in older adults [5].

At the beginning of 2020, the COVID-19 pandemic caused by the SARS-CoV-2 virus [6] produced a global emergency. Governments levied preventive actions such as lockdown, mandatory mask-wearing, and the recommendation of social distancing to diminish the number of infections. The effect of lockdown on the population has been recently analyzed, showing a change in habits in the general population that has negatively affected their health status and wellbeing. Significant increases in psychological, behavioral, and social problems have been reported [7,8], and a decrease in physical activity levels [9] which are correlated with a higher risk of cardiovascular disease, shorter life expectancy, and lower levels of strength and functional capacity in the general population [10,11]. In addition, a study showed that the people who performed vigorous or moderate physical activity during a quarantine reported higher scores in resilience and positive affect and lower depressive symptoms [12]. The reduction in physical activity had a profoundly negative impact on psychological health and wellbeing of a population [13], which was associated with higher anxiety values [14]. From a physiological point of view, inactivity promotes significant losses in muscle size and the contractile properties of muscle fiber, leading to a decrease in strength per unit of cross-sectional area [15]. The rapid deterioration produced by physical inactivity highlights the importance of physical exercise programs even during the pandemic and especially for populations with pathologies, such as persons with sarcopenia. Acknowledging this previous information may be important in developing health- and physical-promotion programs during future periods of confinement.

In this context, patients restarted their training programs after several weeks of lockdown but with the novel mandatory or recommended use (depending on the country) of a mask during training. The aim of the use of face masks is to reduce respiratory droplet excretion from individuals, thereby reducing respiratory virus infections [16]. Surgical and FFP2 masks are the most widely used types of face mask, but they meet different filtration requirements. The FFP2 mask filters small airborne particles and provides less face-seal leakage, so it is more efficacious at reducing viral infections than surgical masks, which only reduce facial contact with large droplets [17]. However, although masks can be effective in reducing virus transmission, mask-wearing is being perceived as inconvenient and uncomfortable and has even resulted in worries that extended mask use might be unhealthy or dangerous, specifically in some cases, such as during exercise.

The effect of face mask-wearing during exercise needs to be discussed. Although some commentaries about the physiological and psychological impact of face mask use have been published [18,19], controversial results have been obtained regarding the use of face masks during exercise. For example, a recent study found that ventilation, cardiopulmonary exercise capacity, and comfort were reduced by surgical masks and highly impaired by FFP2 masks in healthy individuals during an incremental cycling test [20]. However, another study reported that wearing a face mask (cloth or surgical) during vigorous exercise had no discernible detrimental effect on blood or muscle oxygenation nor on exercise performance in young, healthy participants [21]. In addition, no difference in time to exhaustion during an incremental cycling test while wearing surgical or N95 masks compared to a no-mask condition has been found [22]. However, there is no information about the effect of wearing face masks in chronic patients and, specifically, in patients with sarcopenia. In addition, the physiological and performance effects of medical masks during resistance training have never been systematically reported.

It is well-known that resistance training produces less of a cardiorespiratory response than aerobic exercise [23,24,25]. However, in studies involving masks that restrict breathing, the rebreathing of exhaled CO_2_ collected in the dead space of the mask can diminish resistance training performance [26,27,28]. Hence, it seems that limited oxygen availability during exercise and rest intervals when participants wear a mask can affect the muscle’s ability to maintain the balance between ATP breakdown and ATP production, thereby limiting protein C reactive (PCr) recovery, lactate/H+ regulation, and cellular recovery after each exercise bout [29].

Conversely, some studies have suggested that heart rate variability (HRV) can be used as a non-invasive method for assessing autonomic cardiovascular control through the impact of HRV on beat-to-beat heart rate modifications [30,31]. HRV analysis reflects the magnitude of the stress response produced by training [30]. However, to the best of our knowledge, the effect of wearing a surgical or FFP2 mask during a resistance training session on HRV has not yet been analyzed. Therefore, the aim of this randomized cross-over study was to analyze the effect of wearing a surgical or FFP2 mask during a resistance training session on the physiological, perceptual, and strength performance responses in persons with sarcopenia. Considering the previous results regarding the effect of wearing a face mask during exercise [18,19] and that face masks inhibit oxygen uptake and increase carbon dioxide rebreathing [19], our working hypothesis was that wearing a face mask during resistance exercise would impair exercise performance and increase perceptual and physiological demands through a reduction in muscle and blood oxygenation.

## 2. Materials and Methods

A counterbalanced repeated-measures cross-over design was used to determine the effect of wearing a surgical or FFP2 face mask on strength performance and physiological responses during a resistance training session in persons with sarcopenia. The participants completed the following three resistance training sessions with different types of face mask in a randomized order: (1) 4 sets of 10 repetitions of half-squats at 60% of the one-repetition maximum (1RM) and 90 s of rest between sets without a mask; (2) 4 sets of 10 repetitions of half-squats at 60% of 1RM and 90 s of rest between sets wearing a surgical face mask (Krape, Madrid, Spain), and (3) 4 sets of 10 repetitions of half-squats at 60% of 1RM and 90 s of rest between sets wearing a FFP2 face mask (Medi Care System, Barcelona, Spain). Prior to the study, participants read and signed a form to provide informed consent. In addition, the study conforms with the Declaration of Helsinki and was approved by the Catholic’s University of Murcia’s Science Ethics Committee (CE032003).

Fourteen persons with sarcopenia volunteered to participate in the study (*n =* 14 (women: *n =* 10; men: *n =* 4); age: 59.40 ± 5.46 years; weight: 68.78 ± 8.31 kg; height: 163.84 ± 9.08 cm; fat mass: 34.68% ± 4.72%; muscle mass: 42.49 ± 5.04 kg; body mass index (BMI): 25.54 ± 3.96 kg/m^2^). They had been diagnosed with sarcopenia according to the European Working Group on Sarcopenia in Older People (EWGSOP2) criteria [3] by an onboard physician (grip strength <27 kg and <16 kg; appendicular skeletal muscle mass <7 kg/m^2^ and <5.5 kg/m^2^ in men and women, respectively). All participants were involved in a resistance training program for at least the last 3 months. The inclusion criteria were as follows: (1) men and women aged from 50 to 75 years old and with sarcopenia; (2) ability to perform the activities of daily life; and (3) without cognitive impairment. Participants were excluded if they had any, or the absolute or relative contraindication to perform exercise recommended by the American College of Sports Medicine.

Participants visited the laboratory four times. All testing sessions were performed at the same time during the day for each individual and under constant environmental conditions (60% humidity and 20–22 °C). In addition, the control criteria for the participating subjects were to maintain their regular diet and hydration and not to ingest caffeine or alcohol for at least 24 h prior to each training session. Participants were also forbidden from participating in a demanding training session within the preceding 48 h prior to each test. The protocol was explained in the first training session, after which participants signed for informed consent and performed the 1RM testing session after a 10 min standardized warm-up including low-intensity cycling (W45) in a cycloergometer (Technogym, Cesena, Italy) and dynamic mobility. After that, participants performed 1RM based on the movement velocity test for half-squats in a Smith machine (Technogym, Cesena, Italy). In addition, a lineal encoder (Chronojump, Barcelona, Spain) was used to assess the bar’s movement. The load, mean propulsive velocity (MPV), calculated 1RM percentage, estimated 1RM, and the training load calculations (for the next training sessions) were obtained for each participant. The first load was set at 5 kg (the bar without weights); after that, different loads were set, adding 10 kg in each new repetition until the attained MPV was lower than 0.5 m/s, with at least 3 min for resting between repetitions. From that moment, the load was progressively increased in steps of 5 to 1 kg until the 1RM was determined. Two repetitions were performed with light to moderate loads (MPV ≥ 0.50 m/s), but only 1 repetition was performed with heavier loads (MPV < 0.50 m/s). Recovery time was set to 5 min for heavier loads. The estimated 1RM was calculated following previous formulas published elsewhere for the half-squat exercise [32].

After the 1RM testing session, three different sessions in randomized order separated by 48 h were performed during the following days. The strength training session routine involved 4 × 10 repetitions with 90 s rest between sets at 60% 1RM in half-squat with participants either (1) without mask; (2) wearing a disposable surgical mask; or (3) wearing an FFP2 mask.

**Figure 1:**
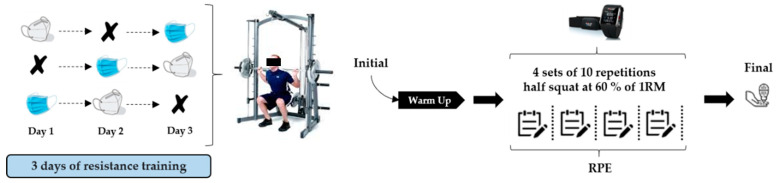
Graphical representation of the study design.

During the entire session, each subject wore an H10 strap Heart Monitor and a Polar V800 heart rate monitor (Polar Electro, Kempele, Finland) to assess HRV. Variables of cardiac autonomic activity were analyzed. The RR series were analyzed using Kubios HRV software (version 2.0, Biosignal Analysis and Medical Imaging Group, University of Kuopio, Kuopio, Finland). The following HRV variables were evaluated: (I) ratio of low frequency (LF) to high frequency (HF) band; (II) total power (TP); (III) percentage of differences between adjacent normal RR intervals > 50 ms (pNN50); (IV) square root of the mean of the sum of the squared differences between adjacent normal RR intervals (RMSSD); (V) standard deviation of all NN normal intervals (SDNN); (VI) natural log of the root mean square difference of successive normal RR intervals (LnRMSSD); and (VII) RR mean intervals. The same Smith machine and lineal encoder were used during the training sessions. Load and MPV were analyzed during each repetition of each session. In addition, ratings of perceived exertion (RPEs) were determined using the 10-point Borg scale [33] following each training session. Capillary blood samples (5 μL) for blood lactate concentration ([Lac]) analysis were collected from a finger prick 2 min after the end of the last repetition and analyzed using a Lactate Pro analyzer (Lactate Pro, Arkay, Inc., Kyoto, Japan) (Figure 1).

Statistical analysis of data was performed with SPSS 25.0 (IBM, Chicago, IL, USA) in the Windows environment. Descriptive statistics with measures of central tendency and dispersion were used. For the inferential analysis, a Shapiro–Wilks W-test was performed to establish the normality of sampling distribution. In addition, a one-way analysis of variance with repeated measures and Bonferroni post hoc were used to investigate differences between study variables. The effect size was calculated using eta-squared (η^2^). For all procedures, a level of significance of *p* ≤ 0.05 was chosen.

## 3. Results

We found no difference in blood lactate concentration, rating of perceived exertion, and strength variables amongst the three evaluated conditions (Table 1).

Regarding heart rate and heart rate variability variables, no significant effect was observed among the three analyzed conditions (Table 2).

Intraclass confident interval (ICC) and coefficient of variation (CV) were: lactate (ICC = 0.96; CV = 10.93%), mean heart rate (ICC = 0.83; CV = 2.57%), mean velocity (ICC = 0.94; CV = 1.35%) and R-R (ICC = 0.87; CV = 2.04%).

## 4. Discussion

The main aim of this study was to evaluate the physiological and strength responses of wearing a disposable surgical mask or an FFP2 mask during a resistance training session in persons with sarcopenia. The main finding is that wearing a surgical or FFP2 mask during a resistance training session did not produce a detrimental effect on strength performance or impact the physiological responses of the body.

To the best of our knowledge, this is the first study to analyze the effect of wearing a medical-grade mask on physiological and strength performance responses during a resistance training session and, specifically, in a population with sarcopenia. Previous studies reported that wearing a mask that restricts breathing (specifically, the Elevation Training Mask^®^) during a resistance training session can diminish strength performance [26,27,28]. In addition, several studies have recently analyzed the effect of wearing a mask during an incremental cycling test [20,21,22] or other less vigorous activities like walking at 2.5 km/h [34], obtaining controversial results. A detrimental effect on cycling performance was observed in healthy adults wearing a surgical or N95 mask [20]. A possible hypothesis to justify this finding is the possible lower arterial hemoglobin saturation produced by a possible decrease in O_2_ consumption and the increase in rebreathing of CO_2_, as suggested previously [19] when the practitioner wears a face mask during exercise. In addition, previous research has shown a decrease in spirometric variables during rest (force vital capacity, peak expiratory flow, or forcer expiratory volume over one second) and progressive cycling exercise (peak ventilatory) in subjects wearing a surgical mask [20], which might indicate higher resistance to breathing. However, our results for strength performance, where no differences among conditions were observed, agree with other studies assessing face masks during progressive-intensity cyclergometry tests [21,22]. These previous studies reported no detrimental effect on exercise performance during a cycling incremental test when the healthy young participants wore a cloth or surgical [21] or a surgical or N95 [22] face mask.

Regarding heart rate and perceptual responses to the resistance training session, our results showed similar responses among mask conditions and the results are in agreement with those of previous studies that compared the effects of wearing cloth [21] or surgical [21,22] face masks during a cycloergometer progressive test. To the best of our knowledge, this is the first study that analyzed the effect of wearing a surgical or FFP2 face mask during exercise on heart rate variability. In the three conditions, the same increase in sympathetic modulation was reflected, showing similar values of RMSSD and SDNN, which are related to sympathetic modulation [35]. Therefore, the cardiac autonomic response was similar during the three-resistance training session. This finding reflects that the three conditions produced the same impact of fatigue on the autonomous response of participants.

Regarding blood lactate concentration, our results suggested that wearing a surgical or FFP2 mask during resistance training sessions produces similar metabolic stress, as evidenced by the similar blood lactate values compared with the no-mask condition. This finding demonstrates that the exercise intensity was similar among conditions, producing the same reliance on anaerobic energy and metabolic acidosis [36]. Our results are not in accordance with a previous study [20] that analyzed the effect of surgical or FFP2 mask-wearing during an incremental cycling test on lactate metabolism, showing a decrease in this marker when the participants wore a mask. These controversial findings could be explained by the type of exercise, as it is well-known that resistance training promotes less of a cardiorespiratory response than aerobic exercise [23,24,25]. Moreover, the training characteristics can be a factor influencing the physiological and performance variables when wearing a mask. In this context, a previous study provided some recommendations about resistance training during COVID-19: training with a lower number of repetitions, longer intervals between sets, and controlled movement velocity to reduce the cardiorespiratory stress and the consequent risk of infection due to the lower pulmonary ventilation and dyspnea [37].

Finally, we acknowledge some limitations of the present study: the sample size was limited, resulting in the low statistical significance of the results. For this reason, our findings cannot be generalized to other types of populations or chronic pathologies. In addition, our results cannot be extrapolated to other types of resistance training (e.g., circuit training) or exercise (e.g., bench press). Concerning the methodological procedures used herein, the lack of assessment of other physiological measurements (e.g., muscle activity by electromyography, muscle saturation by near-infrared spectroscopy, or exercise metabolism measured by gas analysis) that would provide a more comprehensive picture of the mask effect may also be considered a potential limitation. Therefore, physicians, coaches, and practitioners are advised to consider the abovementioned aspects when interpreting the results. From an applied perspective, our findings are important because they indicate that participants, and specifically persons with sarcopenia, can wear face masks during resistance training with no detrimental effect on strength performance or on the physiological response of their body. Therefore, the training dosage and the physiological response during a resistance training session (4 sets of 10 repetitions of half-squats at 60% of the 1RM and 90 s of rest between sets) are not affected if participants wear a face mask. Nowadays, several studies [38,39] have reported the benefits associated with exercise against physiological, social, and psychological effects of COVID-19, recommending performing physical activity. Hence, although wearing a mask produces a feeling of discomfort [20], it seems that the face mask is an appropriate tool to reduce respiratory droplets during indoor exercise (e.g., fitness centers) without affecting the strength performance in terms of physiological responses even in a specific population with a muscle pathology (i.e., sarcopenia).

## 5. Conclusions

Wearing a surgical or FFP2 face mask during resistance training sessions produced similar strength performance and physiological responses as the same exercise without wearing a mask in persons with sarcopenia. This has clinical and practice relevance as COVID-19 severity is associated with many of the risk factors that exercise can improve (e.g., hypertension); hence, exercise should be encouraged in all populations. Therefore, the use of a face mask cannot be an argument to discourage exercise, specifically in persons with sarcopenia during COVID-19, because they can perform exercise using a mask—which is needed to reduce respiratory droplet excretion from individuals and reduce the transmission of respiratory virus infections—with the understanding that the training dosage and physiological response of exercise are the same whether carried out with or without a face mask.

## Figures and Tables

**Table 1:** Blood lactate concentration, rating of perceived exertion, and strength variables results after each training condition.

	Without Mask	Surgical Mask	FFP2 Mask	F	*p*	η^2^
Session Mean	0.396	0.387	0.391	0.138	0.918	0.005
Velocity (m/s)	(0.042)	(0.037)	(0.042)			
Session Peak	0.771	0.771	0.760	0.335	0.846	0.009
Velocity (m/s)	(0.090)	(0.080)	(0.086)			
Session Mean	422.68	412.96	415.72	0.089	0.957	0.002
Power (W)	(84.20)	(85.16)	(96.53)			
Session Peak	881.95	886.32	862.56	0.427	0.808	0.011
Power (W)	(197.40)	(186.40)	(217.26)			
Session Mean	1070.52	1067.87	1077.56	0.008	0.992	0.002
Force (N)	(151.95)	(155.33)	(222.21)			
Session Peak	1282.98	1294.92	1283.16	0.014	0.986	0.007
Force (N)	(199.17)	(203.85)	(240.00)			
RPE (a.u.)	4.12	4.29	4.46	0.697	0.508	0.059
	(0.61)	(0.60)	(0.88)			
Lac (mmol/L)	4.17	5.28	4.49	0.833	0.447	0.073
	(1.89)	(2.07)	(2.45)			

a.u., arbitrary units; RPE, rating of perceived exertion; Lac, lactate.

**Table 2:** Heart rate and heart rate variability results after each training condition.

Variable	Without Mask	Surgical Mask	FFP2 Mask	F	*p*	η^2^
Mean HR (bpm)	123.46	120.54	117.92	0.760	0.479	0.040
	(17.37)	(11.14)	(10.54)			
Peak HR (bpm)	160.92	161.08	157.89	0.436	0.652	0.078
	(21.07)	(17.68)	(10.84)			
RMSSD (ms)	37.69	30.53	30.40	0.532	0.594	0.030
	(21.07)	(16.58)	(21.81)			
SDNN (ms)	66.93	59.85	69.02	0.629	0.542	0.015
	(27.78)	(17.83)	(26.80)			
LnRMSSD (ms)	3.47	3.24	3.14	0.824	0.451	0.030
	(0.60)	(0.68)	(0.80)			
pNN50 (%)	13.23	9.42	9.92	0.437	0.651	0.041
	(12.28)	(7.94)	(10.44)			
Mean RR (ms)	503.79	513.24	526.51	0.573	0.571	0.050
	(63.79)	(48.10)	(46.48)			
Total Power (ms^2^)	1902.64	992.47	2353.68	1.275	0.302	0.018
	(2571.40)	(1059.86)	(3845.95)			
LF/HF	0.98	1.47	1.67	5.47	0.065	0.144
	(0.56)	(0.99)	(0.67)			

HR, heart rate; LF, low frequency; HF, high frequency; pNN50, percentage of differences between adjacent normal RR intervals > 50 ms; RMSSD, square root of the mean of the sum of the squared differences between adjacent normal RR intervals; SDNN, standard deviation of all NN normal intervals.

## Data Availability

No new data were created or analyzed in this study. Data sharing is not applicable to this article.
